# The Iron and Calcium Availability and the Satiating Effect of Different Biscuits

**DOI:** 10.3390/foods12183439

**Published:** 2023-09-15

**Authors:** Antonio Martínez-Martínez, David Planes-Muñoz, Carmen Frontela-Saseta, Gaspar Ros, Rubén López-Nicolás

**Affiliations:** 1 Department of Food Science and Nutrition, Faculty of Veterinary Sciences, Regional Campus of International Excellence “Campus Mare Nostrum”, 30100 Murcia, Spain; 2 Biomedical Research Institute of Murcia (IMIB-Arrixaca), University of Murcia, 30003 Murcia, Spain

**Keywords:** wheat biscuits, mineral availability, phytate, satiety

## Abstract

Biscuits are bakery products made with wheat flour. Wheat is a good source of minerals and dietary fibre, although the presence of phytate or other components could modify mineral availability. In addition, cereal-based products are usually characterised by high fibre content that can influence satiety. The objectives of this study were to evaluate both the iron and calcium availability and the satiety effect of different types of biscuits (traditional recipe vs. “Digestive”) sold in the Spanish market, identifying whether the biscuit type could have effects on these parameters. Nutritional composition and the use of the generic descriptor “Digestive” of biscuits were collected from labels. Phytate and mineral contents were also measured. All samples were previously digested by a simulated process of the gastrointestinal conditions. The satiating effect of biscuits was evaluated according to VAS questionnaires. Results indicated that the mineral content and availability were different between types of biscuits (the traditional recipe showed the highest calcium concentration, while iron was higher in the “Digestive” type). However, mineral availability showed the highest percentages for both minerals, calcium and iron, in the Maria-type samples. Regardless of the different fibre content of both types of biscuits, and despite being higher in the Digestive type than in the Maria type, the satiety measures indicated that the Maria type had more effect on the food intake control. Thus, the descriptor “Digestive¨ in biscuits does not seem to be a marker of better nutritional quality, including parameters of effects on health such as mineral availability or satiety.

## 1. Introduction

Biscuits are a cereal-based foodstuff recipe and are one of the most consumed bakery products due to their variety in texture, flavour and shape and also due to their long storage life. This product is made with several ingredients, wheat flour being the principal one due to its dough formation characteristics. This ingredient is obtained after milling the wheat grain, which is composed of three parts: the reddish colour outer layers called “bran”, the white or yellowish centre “endosperm” and the tiny embryo “germ” [1]. Wheat flour is mainly composed of a mixture of endosperm, starch granules and protein fragments. Among wheat proteins, gliadin and glutenin (gluten) play an essential role in the dough-forming capacity since gluten permits the retention of gas bubbles during baking, producing adequately textured and pleasant eating products [2].

In addition to its dough formation, wheat is considered a good source of protein, minerals, B-group vitamins and dietary fibre [2]. Nevertheless, biscuit processing and the addition of other ingredients (such as sugar and fats) can negatively affect the nutritional quality of these foods. For instance, during milling, bran and germ are separated from starchy endosperm in order to produce white flour, which results in losses of minerals [3].

Minerals are essential micronutrients for human health. Focusing on the most important ones, calcium is required for the formation and maintenance of the skeleton, normal muscle contraction, heart, nervous activity and blood clotting. Iron is incorporated in the haemoglobin molecule and plays a key role in oxygen transport throughout the body. Whole wheat is an important source of minerals essential for health, particularly iron and zinc, and contributes significantly to mineral intake in humans [3,4,5]. Mineral deficiencies of iron, calcium and zinc have a negative effect on human health and may lead to some disease conditions such as iron deficiency anaemia, rickets, osteoporosis and diseases of the immune system [6]. However, to maintain mineral balance, both an adequate intake of a mineral and its overall availability are important [4,7].

Phytic acid (PA or myoinositol hexa-phosphoric acid or IP6) is the major phosphorus storage compound in seeds and cereal grains. It is considered a potent anti-nutrient due to its strong ability to chelate multivalent metal ions, especially iron, calcium and zinc, reducing their availability [8]. It is noteworthy that myoinositol phosphates with less than five phosphate groups (i.e., IP1 to IP4) do not have a negative effect on zinc absorption, whereas myoinositol phosphates with less than three phosphate groups do not inhibit iron absorption [9]. The reduction of phytate content during bakery processing depends on different factors such as phytase activity, degree of flour extraction and dough pH, among others [9]. Therefore, ingredients used in biscuits may have different effects on mineral availability. Likewise, industrial and domestic treatments such as soaking, milling, germination and roasting, as well as alpha-amylase treatment, have been shown to decrease the phytate content of cereals [10].

In addition to the importance of an adequate intake of micronutrients in the prevention of many important diseases, minerals also play an important role in appetite control and energy intake [11,12]. Regarding this, a low consumption of these micronutrients has been related to high body weight and adiposity. Likewise, mineral availability might influence energy balance regulation, and thus, an adequate intake could be associated with lower body weight or even determine the success of weight-reducing programmes [13].

Currently, both the increase in the prevalence of obesity and the obesogenic environment (including the high availability of processed foods) make changes to more healthy habits necessary. These changes should include increasing the choices of healthy foods (many times as substitutes for processed foods). Enhanced satiety foods to promote reduced food intake could be part of this approach and also aid compliance with healthy eating and weight management strategies [14]. Biscuits are included in the ultra-processed foods group according to the NOVA classification [15], and thus, their consumption is related to an increased risk of non-communicable diseases [16]. Specifically, different studies indicate that the ingredients of biscuits are often linked to obesity and overweight [17]. Regarding this, biscuits named “Digestive” usually suggest a beneficial effect on health or a better quality despite that capacity having not been evaluated by EFSA. Biscuits are usually consumed daily and could be a good food item to be used in satiety control. It is important to consider that appetite regulation has numerous determinants, including food composition, digestion, gastric emptying and nutrient absorption, among others. All together, these influence postprandial satiety responses [18]. Another food component involved in satiety is the dietary fibre, which encompasses soluble and insoluble fibres, resistant starches and oligosaccharides. The effect that a specific fibre has on satiety depends on its physical properties, mainly its solubility and viscosity. Several types of viscous fibres increase satiety by increasing stomach distension, which can slow gastric emptying [19]. Thus, one possible solution to design satiating food products is to incorporate highly viscous soluble fibres. However, these fibres generate processing issues that limit their incorporation in food products. Cereal articles, such as biscuits, are good candidates since they consist of concentrated suspensions where these fibres can be dispersed. However, highly viscous soluble fibres have a very high affinity for water. Therefore, during dough mixing, they make a part of the water for the dough creation unavailable. Fibre enrichment requires increasing the water content of the dough, negatively impacting biscuit quality by initiating a gluten network, leading to an increase in biscuit hardness. Thus, it is essential to precisely determine the minimal amount of water to add in order to avoid altering product quality [20].

Given this theoretical framework, the objectives of our study were to evaluate the mineral availability and satiety response of two different types of biscuits: (1) biscuits made from traditional recipes and (2) biscuits labelled as “Digestive”.

## 2. Material and Methods

### 2.1. Samples

Nine different commercial brands of biscuits, including classic biscuit recipes (Maria type) and biscuits labelled as “Digestive”, were purchased from different supermarkets in Spain.

### 2.2. Mineral Content Determination

The iron and calcium concentrations of samples were determined in triplicate by ICP-AES (ICAP 6500 Duo Thermo-One Fast) after the destruction of organic matter with microwave digestion [4]. According to Svecnjak et al. (2013) [21], 500 mg of sample was weighed directly into digestion vessel. A total of 65% HNO_3_ (*v*/*v*) (5 mL) and 30% H_2_O_2_ (*v*/*v*) (2 mL) were added to the samples. Vessels were covered, placed into the rotator body of microwave oven Milestone MLS 1200 Mega Oven (Milestone, Bergamo, Italy) and digestion programme was recalled. Digestion conditions were 1 min at 250 W (smooth oxidation of organic matter), 1 min at 0 W (proceeding of reaction without addition of energy to avoid run-away temperatures and overpressures), 5 min at 250 W (termination of the soft oxidation of the organic compounds), 5 min at 400 W and 5 min at 600 W (final termination of oxidation processes by applying higher power). After cooling, digested samples were transferred to volumetric flasks and diluted to 50 mL using MilliQ water and finally transferred to 50 mL polyethylene flasks. Mineral content of soluble and dialysable fractions was directly measured by ICP-AES.

Availability of minerals in dialysable fraction was determined according to the following formula [22]: dialysis mineral (%) = (dialysis mineral concentration × dialysis ml)/(total mineral concentration) × 100.

### 2.3. Inositol Phosphates Content

IP content was extracted from samples according to Frontela et al. (2011) [9]. Fat was removed from 5 g of samples with 5 mL of hexane and centrifugation to 4500× *g* for 20 min. Supernatant was discarded, and samples were dried and stored at −20 °C until extraction.

Inositol phosphates were extracted from 0.5 g of sample and 10 mL of 0.5 M HCl with gently shaking at room temperature for 2 h. Then, the extract was centrifuged to 4500× *g* for 20 min, and the supernatant was frozen (−20 °C) overnight. The next day, samples were thawed and centrifuged to 4500× *g* for 5 min. A total of 1 mL aliquot of supernatant was diluted with deionised water and poured onto an anion exchange (SAX) column (500 mg; Supelco, Bellefonte, PA, USA) that was connected to a vacuum manifold set at 20 mmHg. The column was previously washed with 0.05 M HCl to remove inorganic phosphates present in the resin. The resin-bound IPs were then eluted with 2 mL of 2 M HCl and then evaporated to dryness in a rotary evaporator at 40 °C and dissolved in 1 mL of deionised water containing 15 µL tetrabutylammonium hydroxide (Sigma-Aldrich, Steinheim, Germany).

IPs were analysed in quadruplicate by HPLC/MS system consisting of an Agilent 1100 Series HPLC (Agilent Technologies, Santa Clara, CA, USA) equipped with a thermostatic µ-wellplate autosampler and a quaternary pump and connected to an Agilent Ion Trap XCT Plus Mass Spectrometer (Agilent Technologies, Santa Clara, CA, USA) using an electrospray (ESI) interface. Samples were passed through 0.22 µm HPLC filters before injection. Mobile phase A consisted of MilliQ water + 0.1% formic acid, and mobile phase B consisted of Acetonitrile + 0.1% formic acid, which were used for the chromatographic separation. The mass spectrometer was operated in the polarity negative mode with a capillary spray voltage of 3500 V and Ultra Scan speed of 26,000 (m/z/seg), with a target mass of 650 m/z. The nebuliser gas pressure, drying gas flow rate and drying gas temperature were set at 30 psi, 8 L/min and 350 °C, respectively. Control and data acquisition of the HPLC-MS equipment were performed with Agilent Chemstation Rev. B.01.03. SR2. Data were processed using the Data Analysis software for LC/MSD Trap Version 3.3 (Bruker Daltonik, GmbH, Bremen, Germany) provided by the manufacturer. The molar ratios of phytate to iron and calcium were calculated as the millimol of phytate present in the sample divided by the millimol of iron and calcium present in the sample, respectively.

### 2.4. In Vitro Digestion

Static in vitro digestion modelling was performed using the method of Minekus et al. (2014) [23] with some modifications. Firstly, simulated digestion fluids were prepared (Simulated Salivary Fluid (SSF), Simulated Gastric Fluid (SGF) and Simulated Intestinal Fluid (SIF)). Simulated in vitro digestion process consisted of three phases: oral, gastric and intestinal. In oral phase, 2 g of biscuit homogenised with 5.5 mL of deionised distilled water and 7 mL of SSF electrolyte stock solution (plus CaCl_2_ to achieve 0.75 mM) were mixed and adjusted to pH 7. Then, 0.5 mL of human salivary alpha-amylase 75 U mL^−1^ (10.5 mg) was added, and the sample was incubated for 2 min in a shaking water bath at 37 °C and 120 strokes/min. For gastric digestion, 14.5 mL of SGF (plus CaCl_2_ to achieve 0.075 mM) was added. Then, pH was adjusted at 3 with 6 M HCl, and 0.5 mL of porcine pepsin 2000 U mL^−1^ (58 mg) was added. It was incubated for 2h in the same conditions (temperature and shaking) as described before. In the intestinal phase, 29 mL of SIF (plus CaCl_2_ to reach 0.3 mM) were added to mixture and pH was adjusted to 7. Then, 0.5 mL of pancreatin 100 U mL^−1^ (465.52 mg) and 0.5 mL bile salts (496.55 mg) were added. From the total volume, aliquots of 20 mL were taken for soluble and dialysable fractions. The first one was incubated for two hours; meanwhile, for dialysis, a dialysis membrane with a pore size (MMCO) of 12,000 Da (dia. inf. 36/32 in. to 28.6 mm, 30 m, Medicell Int. Ltd., London, UK) was filled with 12.5 mL of SIF, which was included in the tube and incubated for 2 h as before. After the incubation, soluble fraction was centrifuged (Eppendorf 5804-R Centrifuge, Hamburg, Germany) for 10 min to 760 g, and the supernatant was collected and stored at −80 °C until analysis, similar to the dialysis membrane content.

### 2.5. Evaluation of Satiety

Twenty-five volunteers (10 men and 15 women) were recruited for the study, which followed a random design of two different breakfasts that included Maria-type or Digestive-type biscuits. The number of participants was calculated using the following formula:

$$n=\frac{2*{\left(Za+Zb\right)}^{2}*S^{2}}{d^{2}}$$

where *Za* = study safety (1.96), *Zb* = statistical power (0.2), *S* = variance (18,000,000) and *d* = magnitude of difference (2000).

The inclusion criteria were the following: aged between 22 and 49 years (mean ± SD: 35.5 ± 13.5), body mass index (BMI) between 20 and 30 kg/m^2^ (mean ± SD: 25 ± 5) and stable body weight (body weight change < 5 kg for three months before screening). The exclusion criteria were the following: smoking (more than five cigarettes per day), daily use of prescription medication (except for hypertension medications and oral contraceptives), pregnancy or lactation and body mass index (BMI) > 30 kg/m^2^ [11]. At screening, volunteers received all information concerning the study, including the objective, design, duration, any risks and the benefits of the study. All participants filled out an informed consent form. The study was approved, according to the Declaration of Helsinki, by the Bioethics Committee of the University of Murcia with the code 2051/2018.

Different breakfasts of approximately 325 kcal each were provided to participants with a washout period of approximately one week. Both test meals consisted of 150 mL of semi-skimmed milk and 50 mL of natural coffee (80 kcal). In one type of them, the participants received approximately 55 g of Maria-type biscuits (245 kcal); meanwhile, the other type of breakfast consisted of 51 g of Digestive-type biscuits (245 kcal). The energy content of the test meals was individualized to contain approximately 25% of total energy requirements. Participants were instructed to eat their habitual dinner prior to the day of the breakfast and fast for at least 12 h. They were also asked to abstain from alcohol and vigorous exercise for 24 h prior to each test.

Satiety of the different types of biscuits was determined by Visual Analogue Scale (VAS) (100 mm lines) of the means of seven surveys. Each participant completed six identical surveys with 11 questions about appetite and one survey with 17 different questions about appetite and palatability through a slider (from “nothing” to “extremely or very much”) using validated software (Satin APPetite software version 1.0) installed on their mobile devices or tablets. The first survey was filled in before breakfast (time 0), and the remaining surveys at 15, 45, 75, 105, 135 and 165 min later. The incremental area under the curve (AUC) of the most representative appetite VAS scores was measured by the trapezoidal method [18,24]. Furthermore, the VAS score for peak–nadir was also calculated by subtracting the lowest score from the highest score recorded during the meals.

### 2.6. Statistical Analysis

The normal distribution of the data was verified using the Shapiro–Wilk test. The homoscedasticity was checked using Bartlett test when the data were normal and Levenne test when the data were not normal, whilst homoscedasticity VAS analysis was checked using F of Snedecor test. The minerals and inositol phosphates content, dialysis fraction of the minerals and satiety between sample biscuits were performed by one-way ANOVA with a subsequent HSD Tukey post hoc. To compare biscuits regarding mineral contents and dialysis fraction, Wilcoxon rank sum exact test was used; meanwhile, for inositol phosphates content, the robust method for trimmed means was used. Statistical analysis of the data was carried out using a statistical software package RStudio for Windows (version 1.0.143) with a significant level of *p* < 0.05. Results were expressed as mean ± SD.

## 3. Results and Discussion

Biscuits are small bakery products usually made from short dough with added sugar and fats and are relatively low in water. Moreover, some minor ingredients, such as sodium bicarbonate, ammonium bicarbonate and emulsifiers, to improve the colour, flavour, texture and consistency are also added [1]. Table 1 shows the ingredients of different commercial brands of two types of biscuits (Digestive (A, B, C, D, E and F) and Maria type (G, H and I)) used in the present study.

As can be observed, the main ingredient for all biscuits was wheat flour, except for Digestive A, which was oatmeal. All Digestive biscuits contain whole wheat flour in different percentages except Digestive D, which contains wheat flour, similar to the Maria type. In addition, a very low amount of whole wheat flour in Digestive type A and F is mentioned (14.1–17.5%) compared to the other Digestive-type biscuits (approximately 60%). Furthermore, only Digestive D biscuit reported no sugars added, using added sweeteners instead.

All the biscuit types contained vegetable oils and fats from different origins and with different proportions (≤19%), and two brands of the Digestive type contained palm fat as the only fat added. According to different studies, cereals and cereal products are important dietary sources of different minerals [25], providing an average of 31% of calcium and 39% of iron in the diet. The two different breakfasts designed for the present study provided an average of 9 mg of calcium and 0.8 mg of iron per breakfast in the case of Digestive biscuits; meanwhile, in the case of the Maria type, each breakfast provided an average of 12.9 mg of calcium and 0.6 mg of iron.

These micronutrients exert key functions in the macronutrient synthesis and in human physiology [4]. In our study, as can be observed in Table 2, the Digestive-type biscuit (A) (containing oatmeal) showed the highest concentration of iron (2.36 mg/100 g). Regarding calcium, different biscuits (two brands of Maria type and two brands of Digestive type) showed values >20 mg/100 g. These results are probably due to the presence of oatmeal, which has been reported to have more iron and calcium content than wheat, according to the Spanish Food Composition Database Network [26]. In addition, the mineral content of biscuits containing whey powder or skimmed milk powder showed the highest calcium concentration (including both Digestive and Maria types) despite their elaboration with refined wheat flour.

Values of energy, nutrients and salt can be seen in Table 3, as reported by the label. According to the results, a low variability among the different biscuits was observed. The total median energy was 487 and 455 Kcal/100g for Digestive and Maria types, respectively. The amount of sugar (g/100 g) in the Digestive type was lower than values observed in the Maria type.

Whole grain flour represents a good source of minerals; meanwhile, the refined processing of wheat flour supposes a loss of micronutrient concentrations [27]. Furthermore, as mentioned before, oat flour contains considerable amounts of valuable nutrients, including iron and calcium [28]. However, oat flour also has been previously reported to have a high content of phytic acid (>1100 mg/100 g) [22].

Regarding the anti-nutrient compounds of biscuits, the hexa- (IP6) and penta- (IP5) forms of IP account for over 90% of the total phytate content in raw grains. These forms give rise to insoluble salts of iron, calcium and zinc with poor absorption characteristics and, hence, low availability. A reduction of IP6 and IP5 forms to lower levels is highly desirable in the industrial production of cereal products in order to reduce the negative effects of phytate (IP5 + IP6) on mineral availability.

According to our results, presented in Table 4, the Digestive type showed statistically significant (*p* < 0.05) higher values of phytate than those observed in Maria-type biscuits, ranging between 551 and 674 mg/100g, except for the Digestive biscuits mainly containing wheat flour. Two types of the Digestive type presented a similar content of phytate (IP5 + IP6) than those observed in the Maria type, probably due to its low concentration of whole wheat flour as an ingredient.

The phytate content of bakery products made from whole wheat flour is higher than those observed in products made with wheat flour [9,29], which may impair mineral absorption. Regarding this, the estimation of the dietary phytate-to-mineral molar ratio is a valuable tool in predicting the inhibitory effect of phytate on the in vitro mineral availability [8,30,31,32,33]. According to these references, mineral availability could be committed with a ratio of phytate:iron and phytate:calcium higher of 0.4:1 and 0.24:1, respectively. Table 4 lists the molar ratios of phytate to iron and calcium of biscuits. Considering that biscuits made with wheat flour or very low amounts of whole wheat flour showed a mean of 0.065 and 1.34 for the molar ratios of phytate to calcium and iron, respectively. In the case of the Maria type, low molar ratios of phytate to iron were found (0.4–1:1). However, in all biscuits, observed values can compromise the absorption of iron. For calcium, we observed that biscuits including whole wheat flour or oatmeal as the first ingredient compromised mineral availability, achieving values > 0.24:1 (ratio phytate: calcium). However, despite Maria-type biscuits containing important amounts of wheat flour, the estimated availability of calcium was not compromised.

The next step in our study was to determine the in vitro mineral availability of biscuits. Mineral solubility (%) and dialysis (%) have been widely employed in the literature to study in vitro mineral availability [4,22]. The method consisted of simulating gastrointestinal digestion and measurement of the mineral solubility and dialysis percentages. This identifies the amount of minerals available in the gastrointestinal tract for absorption [5]. Table 5 shows differences in the percentages obtained for the dialysis fraction of calcium and iron of the different biscuits. As can be observed, most biscuits made with whole meal flour or oatmeal showed the lowest percentages of dialysis of both minerals iron and calcium in a statistically significant manner. Overall, the Maria type showed percentages of dialysis >25% and >1.55% in calcium and iron, respectively. In the case of iron, values were aligned with results observed in other cereals products [22]; for calcium, the biscuit (Digestive type) with the lowest phytate-to-mineral molar ratio showed a high percentage (<28%) of calcium dialysis, similar to other foods not containing phytate [34]. In Table 5, it is observed that biscuits containing whey powder or milk showed a similar percentage of iron dialysis to other types of biscuits. This could be due to the high concentration of refined wheat flour (with low concentrations of phytate) compared to the Digestive type, suggesting that the inhibitory capacity of phytate on the iron availability is stronger than that exerted by calcium, casein or milk proteins [22]. Moreover, the food matrix and ingredients of bakery products can largely modify mineral absorption [9]. Regarding this, it should also be considered that the bakery process using different raising agents between Digestive and Maria types could contribute to differences in mineral availability from the matrix [35]. In terms of labelling, the biscuits of this study cannot be considered dietary sources high in either iron or calcium; however, considering the mineral availability of biscuits, they can be considered a good option to provide calcium and iron in the diet.

As expected, the phytate–mineral molar ratios are an adequate tool to predict mineral absorption [22] since biscuits with the lowest phytate-to-mineral molar ratios showed the highest mineral dialysis percentages.

As mentioned before, minerals are essential for the proper development and function of the human body, preventing anaemia and other important diseases [6] and, therefore, have to be provided in the daily diet. Moreover, according to different studies, minerals can also play an important role in appetite control and energy intake [11,36]. This viewpoint seems to be consistent due to the low consumption of these micronutrients, which has been shown to be inversely associated with body weight and adiposity in many studies [13,37]. In fact, there are multiple pathways by which micronutrient deficiencies could impair appetite regulation and energy metabolism despite specific pathways being poorly understood [37]. Major et al. (2008) [38] found that vitamin–mineral supplements had a beneficial effect on appetite regulation suggesting a role of micronutrients in satiety. Therefore, it would be key to consider the importance of the availability of minerals in the energy balance regulation. Then, an adequate intake of these micronutrients could be associated with lower body weight or even determine the success of weight-reducing programmes [11].

Appetite is modified due to variations in hormone levels implicated in the regulation of energy balance, such as insulin, leptin and cortisol. Minerals are involved in the synthesis of these and other peptides and neurotransmitters that control food intake, so a mineral deficiency could affect peptide hormone levels and thus interfere with the signalling pathways that control food intake [18]. Studies in human volunteers have shown that inhibition of glucose utilisation and fatty acid oxidation resulted in an increased appetite and energy intake, inducing a decrease in blood glucose concentrations [24]. Minerals are essential to energy substrate oxidation and must be supplied by the diet; thus, a lower availability could result in signalling to feeding centres to increase energy intake in order to satisfy the mineral requirements [39].

According to the nutritional composition provided by the label of each biscuit type, energetic values were similar between samples, with slight differences ranging between 440 and 523 kcal/100 g (Table 3). A similar trend was observed for the protein (values between 7.6 and 6 g/100 g) and fibre contents, except for Digestive A (the only biscuit containing oatmeal), which showed the highest concentration of fibre (6.8 g/100 g) compared to the others ranging between 4.3 and 2.1 g/100 g. Thus, differences observed in satiety seem not to be due to differences in macronutrient contents or energetic values of biscuits. Recent studies [40] indicate that micronutrient composition influences food choice, and thus, preferences can be modified after eating mineral-rich foods. According to our results, not only minerals or macronutrient compositions are responsible for the satiety of biscuits. Regarding this, in our study, biscuits containing oatmeal (Digestive type) showed low percentages of mineral dialysis despite the mineral content of both iron and calcium being high; this low availability was mainly due to its high phytate content (IP5 + IP6). Oatmeal also provides biscuits with beta-glucans and the highest viscosity, and although it has been reported to not impair mineral absorption [41], there are many studies indicating that oatmeal exerts a complex and inconsistent effect on satiety [42].

**Figure 1:**
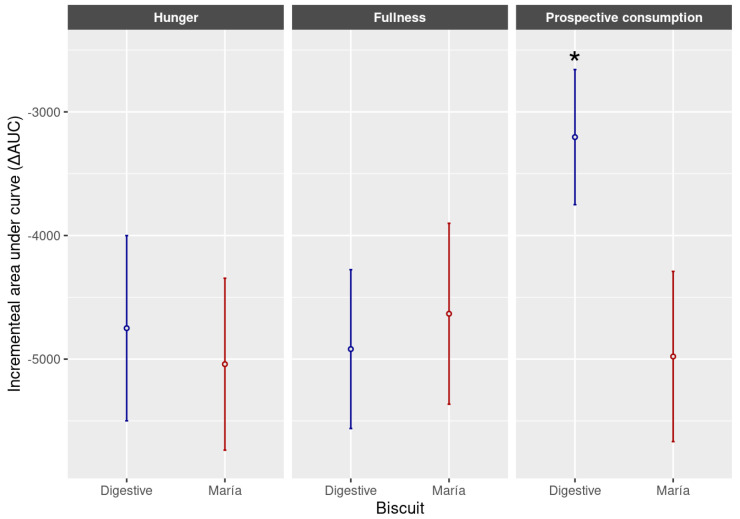
Incremental area under the curve (ΔAUC) of hunger, fullness and prospective consumption of Digestive and Maria biscuit types. Values are expressed as means ± SD. * indicates statistically significant differences (*p* < 0.05).

**Figure 2:**
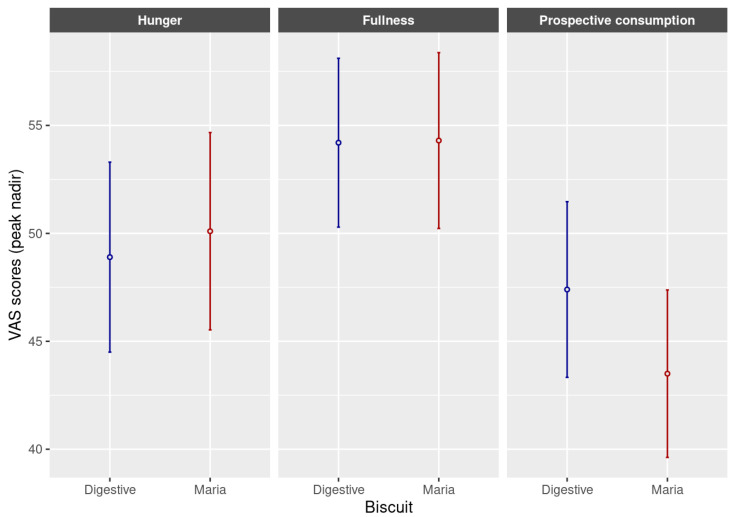
Peak–nadir of hunger, fullness and prospective consumption of Digestive and Maria biscuit types. Values were expressed as mean ± SD.

The present study collected data on the phytate content, mineral availability and satiety of biscuits labelled as “Digestive” and traditional biscuits labelled as “Maria”. Two useful indicators to determine appetite, measured by VAS score along the time, are the incremental AUC and peak–nadir [43] since these parameters are not affected by the baseline values (fasting values) marked by volunteers in the different assayed tests. Results obtained for AUC and peak–nadir are shown in Figure 1 and Figure 2, respectively. According to our results, surprisingly, despite one type of Digestive biscuit containing oatmeal and a higher fibre proportion, this type of biscuit did not show the highest satiety through the hunger and fullness feelings reported by participants through VAS surveys (Figure 1). On the contrary, as it can be observed in Figure 1, the prospective consumption marked by volunteers (amount of food that they wanted to eat during the study time) was statistically lower (*p* < 0.05) in the case of Maria-type biscuits than Digestive ones. The declared compositions of both types of biscuits were very similar. As can be observed, the most noticeable differences were the fibre content and values observed on mineral availability. Despite the Digestive biscuits being the ones providing the greatest concentrations of fibre, the Maria biscuit type showed a significantly lower sensation of prospective consumption that could be explained by the greater availability of minerals observed in this type of biscuit. Traditional biscuits labelled as “Maria type” exhibited higher in vitro availability of both minerals iron and calcium than biscuits labelled as “Digestive type”. These findings could be explained because, in our study, traditional biscuits induced more satiety. Regarding this, there are studies attributing an effect of micronutrients on the control of food intake [13].

The authors are aware of several limitations of the present study. Biscuits are complex formulations with different amounts of ingredients that complicate the collection of fair conclusions. The main limitation to highlight, among others, is the presence of different ingredients in commercial biscuits, even when considering the same type of biscuit. These differences are probably modifying the expected satiety response of volunteers and hinder the obtaining of accurate results. Although these limitations reduce the strength of conclusions, the present study could provide support for the benefits of the mineral availability of biscuits on satiety. Then, it could be desirable to produce biscuits with improved mineral availability and satiety capacity based on the reduction of refined wheat flour. Despite oatmeal providing important functional components, it seems not to be key for satiety when compared with types of biscuits containing wheat. Considering the popularity of biscuits, this information could be valuable for a more healthy and nutritive formulation of this type of highly consumed ultra-processed food. Future research should consider counterbalancing the complexity of biscuits for a better knowledge of the effect of their mineral availability on satiety.

## Figures and Tables

**Table 1:** Biscuit ingredients.

Biscuits	Ingredients
Digestive A	Oatmeal 43.5%; whole wheat flour 17.5%; vegetable oil (high oleic sunflower) 17.5%; sugar, glucose and fructose syrup; raising agents (sodium and ammonium carbonate); salt
Digestive B	Whole wheat flour 59%, vegetable fat (palm), sugar, glucose and fructose syrup, raising agents (sodium and ammonium carbonate), salt
Digestive C	Whole wheat flour 62%; vegetable fat (palm); sugar, glucose and fructose syrup; raising agents (sodium and ammonium carbonate); salt
Digestive D (0% sugar)	Wheat flour *; vegetable oils (high oleic sunflower and palm); sweeteners (maltitol, maltitol syrup, sucralose); wheat bran; fructooligosaccharides syrup; raising agents (sodium and ammonium bicarbonates); salt; antioxidants (E 304, E 306) and acidity regulator (potassium tartrate)
Digestive E (33% reduced fat)	Whole wheat flour 64.5%; sugar; high oleic sunflower oil 13.5%; glucose and fructose syrup; raising agents (sodium and ammonium carbonate); salt
Digestive F	Cereals 68.8% (wheat flour 54.7%, whole wheat flour 14.1%); sugar; high oleic sunflower oil 12.7%; palm oil; sugar syrup; raising agents (sodium, malic and ammonium carbonate); fructooligosaccharides syrup; salt; emulsifiers (soy lecithin, sunflower lecithin)
Maria G	Wheat flour 74.3%; sugar; palm fat; glucose and fructose syrup; whey powder; raising agents (sodium and ammonium carbonate); salt; emulsifiers (soy lecithin, sunflower lecithin); sodium metabisulfite; scent
Maria H	Wheat flour 66%; oil-free 19% (vegetable oils of high oleic sunflower and palm); sugar; whey powder; glucose and fructose syrup; raising agents (sodium and ammonium carbonate); salt; emulsifiers (lecithin); scent; sodium metabisulfite
Maria I	Wheat flour 59%; sugar; vegetable oils (soy, corn, high oleic sunflower) 9%; glucose syrup; skim milk powder; palm fat; pea fibre; salt; emulsifiers (sunflower lecithin, E472e); raising agents (sodium and ammonium carbonate); antioxidants (sodium metabisulfite, E304, E306); scent; vitamins (B6, folic acid, B12)

* Proportion not specified by the manufacturer.

**Table 2:** Total mineral content (per 100 g).

Biscuits	Ca (mg/100 g)	Fe (mg/100 g)
Digestive A	21.53 ± 7.59 ^ab^	2.36 ± 0.60 ^a^
Digestive B	12.80 ± 0.10 ^b^	1.69 ± 1.11 ^ab^
Digestive C	21.00 ± 4.85 ^ab^	1.27 ± 0.08 ^ab^
Digestive D	19.40 ± 6.86 ^ab^	1.47 ± 0.27 ^ab^
Digestive E	14.60 ± 1.90 ^b^	1.41 ± 0.20 ^ab^
Digestive F	18.03 ± 6.37 ^ab^	1.26 ± 0.08 ^ab^
María G	15.87 ± 3.38 ^b^	1.36 ± 0.24 ^ab^
María H	24.53 ± 2.51 ^ab^	0.91 ± 0.10 ^b^
María I	29.73 ± 2.02 ^a^	1.24 ± 0.02 ^ab^

The results are shown as mean ± SD of triplicate determinations. (^a,b^) Different letters in superscript within the same column indicate statistically significant differences (*p* < 0.05).

**Table 3:** Nutritional composition of different biscuits (values per 100 g of biscuit).

Nutritional Information	Digestive A	Digestive B	Digestive C	Digestive D	Digestive E	Digestive F	María G	María H	María I
Energetic value (kcal)	477	483	483	523	477	480	440	482	442
Total fat (g)	21	21	21	21	14	20.50	10.50	20	11
SFA (g)	2.30	10	10	4.10	1.50	4.70	5.10	4.20	2.20
MUFA (g)	16	-	-	-	11	-	-	13	-
PUFA (g)	2.70	-	-	-	1.50	-	-	2.80	-
Carbohydrates (g)	61	66	66	81	72	64	77	68	77
Sugars (g)	18	18	18	<0.50	18	18	24	21	24
Dietary fibre (g)	6.80	3.00	3.00	3.10	3.70	4.30	2.10	-	3.50
Proteins (g)	7.50	6.00	6.00	6.10	6.50	7.10	7.60	6.40	7.00
Salt (g)	1.10	1.60	1.60	1.80	1.60	1.68	0.83	0.80	1.00

**Table 4:** Inositol phosphates content (mg/100g) and molar ratios of the biscuits.

Biscuits	IP5 (mg/100 g)	IP6 (mg/100g)	IP5 + IP6 (mg/100 g)	IP5 + 6:Ca	IP5 + 6:Fe
Digestive A	361.48 ± 0.69 ^b^	193.42 ± 1.64 ^b^	554.90 ± 1.00 ^b^	1.6:1	21.6:1
Digestive B	355.88 ± 3.18 ^b^	195.52 ± 4.15 ^b^	551.40 ± 5.31 ^b^	2.7:1	28.8:1
Digestive C	384.70 ± 6.86 ^a^	289.35 ± 3.87 ^a^	674.04 ± 0.01 ^a^	2:1	45.7:1
Digestive D	44.13 ± 0.19 ^c^	23.48 ± 0.16 ^d^	67.61 ± 0.09 ^d^	0.22:1	4.07:1
Digestive E	27.98 ± 0.43 ^d^	130.33 ± 0.87 ^c^	158.30 ± 1.29 ^c^	0.66:1	9.76:1
Digestive F	4.62 ± 0.14 ^e^	4.73 ± 0.08 ^e^	9.34 ± 0.20 ^e^	0.032:1	0.61:1
María G	31.14 ± 0.13 ^cd^	31.84 ± 1.06 ^d^	62.98 ± 1.17 ^d^	0.03:1	0.45:1
María H	2.37 ± 0.10 ^e^	2.60 ± 0.10 ^e^	4.97 ± 0.02 ^e^	0.013:1	0.5:1
María I	7.14 ± 0.05 ^e^	7.21 ± 0.15 ^e^	14.35 ± 0.10 ^e^	0.03:1	1.06:1

The results are shown as mean ± SD of quadruplicate determinations. (^a–e^) Different letters in superscript within the same column indicate statistically significant differences (*p* < 0.05).

**Table 5:** Dialysis fraction (%) of calcium and iron of the biscuits.

Biscuits	Dialysis Ca (%)	Dialysis Fe (%)
Digestive A	11.15 ± 0.96 ^d^	1.89 ± 0.52 ^c^
Digestive B	10.85 ± 6.61 ^d^	1.77 ± 1.38 ^c^
Digestive C	12.96 ± 2.82 ^d^	1.06 ± 0.69 ^c^
Digestive D	23.82 ± 2.87 ^b^	2.01 ± 0.22 ^c^
Digestive E	12.45 ± 2.76 ^d^	2.33 ± 0.27 ^b^
Digestive F	28.37 ± 1.14 ^b^	1.57 ± 0.64 ^c^
María G	52.31 ± 3.24 ^a^	2.75 ± 0.02 ^b^
María H	35.33 ± 0.78 ^b^	3.42 ± 0.12 ^a^
María I	25.01 ± 1.03 ^b^	1.55 ± 1.17 ^c^

The results are shown as mean ± SD of triplicate determinations. (^a–d^) Different letters in superscript within the same column indicate statistically significant differences (*p* < 0.05).

## Data Availability

The data used to support the findings of this study can be made available by the corresponding author upon request.

## Abbreviations

HPLC-MS, high-performance liquid chromatography–mass spectrometry; ICP-AES, inductively coupled plasma–atomic emission spectrometry; VAS, visual analogue scale; PA, phytic acid; W, watts; V, volts; SSF, simulated salivary fluid; SGF, simulated gastric fluid; SIF, simulated intestinal fluid; AUC, area under the curve; SD, standard deviation; IP, inositol phosphates; RDA, recommended dietary allowances.
